# Analysis of the Monophyletic Lineage of Avian Influenza H5N1 Which Circulated in Venezuelan Birds During the 2022–2023 Outbreak

**DOI:** 10.3390/microorganisms12122519

**Published:** 2024-12-06

**Authors:** Carmen Luisa Loureiro, Valeria Bonetti, Rossana C. Jaspe, Yoneira Sulbaran, Wilmer Alcazar, Carlos Hernández, Nardraka Rodríguez, Hector R. Rangel, Jose Luis Zambrano, Flor H. Pujol

**Affiliations:** 1 Laboratorio de Virologia Molecular, Centro de Microbiología y Biología Celular (CMBC), Instituto Venezolano de Investigaciones Científicas (IVIC), Caracas 1020, Venezuela; cloureir1@gmail.com (C.L.L.); valeriabonetti@gmail.com (V.B.); rossanajaspesec@gmail.com (R.C.J.); yfsulbara@gmail.com (Y.S.); hrangel2006@gmail.com (H.R.R.); 2 Instituto Nacional de Salud Agricola Integral (INSAI), Maracay 2101, Venezuela; wilmeralcazar@hotmail.com (W.A.); saludaviarinsai@gmail.com (C.H.); nardrakar@hotmail.com (N.R.); 3 Laboratorio de Virologia Celular, Centro de Microbiología y Biología Celular (CMBC), Instituto Venezolano de Investigaciones Científicas (IVIC), Caracas 1020, Venezuela

**Keywords:** avian influenza, South America, clade 2.3.4.4b, mortality, avian receptor

## Abstract

Avian influenza subtype H5N1 has caused outbreaks worldwide since 1996, with the emergence of the Guandong lineage in China. The current clade 2.3.4.4b has evolved from this lineage, with increased virulence and mass mortality events in birds and mammals. The objective of this study was the analysis of 17 viral genomes of H5N1 avian influenza isolated in Venezuela during the 2022–2023 outbreak. The eight viral genomic segments were amplified using universal primers and sequenced via next-generation sequencing. The sequences were analyzed to confirm the H5 hemagglutinin clade, identify possible genetic reassortments, and perform a phylogenetic and docking analysis of the viral isolates. The viruses found in Venezuela belonged, as expected, to clade 2.3.4.4b and formed a monophyletic clade with North American influenza viruses, with no evidence of further reassortment. The introduction of the virus in South America is associated with bird migration through the Atlantic (Venezuela), Atlantic/Mississippi (Choco, Colombia), and Pacific migratory flyways, with the emergence of several viral lineages. Several mutations were found in all segments of the genome, although none of the key mutations was involved in mammalian adaptation. Moreover, in silico structural analysis suggests, as expected, that the viral hemagglutinin maintained a predilection for avian α2,3-linked sialic acid. The unprecedented pathogenic outbreak of avian influenza disease in South America was associated with the circulation of three different lineages, which maintain a lower affinity for the mammalian receptor.

## 1. Introduction

Avian influenza is a virus of the *Orthomyxoviridae* family. It spreads mainly among wild waterfowl but can also be transmitted to mammals and humans, causing serious symptoms and in many cases a fatal outcome [1]. Avian influenza subtype H5N1 has caused outbreaks worldwide since 1996, when the first outbreak of highly pathogenic avian influenza (HPAI) was detected in Guangdong, China [2].

The evolution of the HPAI subtype H5N1 A/goose/Guangdong/1/1996 lineage resulted in the divergence and generation of 10 distinct virus clades (0 to 9) and multiple subclades [3]. Initially, the H5N1 A/goose/Guangdong/1/1996 lineage virus was restricted to southern China. In 2003, it began to spread to other Asian countries and, by 2008, had disseminated to more than 60 countries [4]. In addition, cases of transmission of viruses from birds to humans have been reported [5]. Through multiple reassortments with low pathogenic avian influenza (LPAI) viruses, HPAI viruses acquired new combinations of internal genes, and ultimately, clade 2 proved to be more successful [6]. Between 2009 and 2013, H5Nx HPAI viruses of clade 2.3.4 showed an apparent geographic expansion, also including other subtypes in addition to H5N1 [4].

The current clade 2.3.4.4b has evolved from the Guangdong virus lineage but has increased its virulence and generated mass mortality events in birds and mammals on an unprecedented scale [2]. This clade of HPAI virus has been spreading since 2020, predominantly via migratory birds to Africa, Asia, and Europe, since 2021 in North America, and then South America in October 2022 [7]. These HPAI viruses exhibit a furin cleavage site in the hemagglutinin, which allows its rapid dissemination throughout the body [1]. They also have a constellation of genes that has adapted over the years, which allows them to infect their host with great efficiency.

Several human cases have been reported from the influenza H5N1 clade 2.3.4.4b, many of the most recent infected with the B3.13 genotype within this clade, circulating among cows in the USA [7,8]. This viral outbreak and spillover events in a broad host range are concerning and may suggest increasing virus adaptation in mammals. Genomic surveillance of this influenza virus is crucial to detect its evolution and to prevent cross-species transmission [7,8].

The first cases of influenza H5N1 in Venezuela were detected in two Pelicans on 25 November 2022, and cases were detected up to the end of January 2023. The aim of this study was the molecular characterization of 17 viral genomes of H5N1 avian influenza isolated in Venezuela during the 2022–2023 outbreak and its comparison with the other South American isolates.

## 2. Materials and Methods

### 2.1. Real-Time PCR Detection of Influenza H5N1 Samples

This study was approved by the Animal Bioethical Committee of IVIC (COBIANIM2023-03). Viral RNA was extracted from tracheal or cloacal swabs with a QIAamp Viral RNA Mini kit (QIAGEN, Hilden, Germany). Reverse transcriptase real-time PCR (qRT-PCR) was performed using previously reported procedures for H5 [9] and N1 [10].

### 2.2. Genome Amplification and Sequencing

Complete genome amplification was performed according to Zhou et al. [11]. Since the first genomic fragments of influenza viruses could not be sequenced for some isolates, primers specific for these 3 segments were designed. For segment 1, FPB2 (5′-ctaatgtcacagtctcgcactc-3′) and RPB2(5′-caagattgctcagctcattg-3′) were used as forward and reverse primers, respectively, for segment 2, FPB1(5′-tgccataagtaccacattcc-3′) and RPB1(5′-catgaaggacaagctaaattcac-3′) were used, and for segment 3, FPA(5′-atggaagactttgtgcgac-3′) and RPA (5′-ctatttcagtgcatgtgtgagg-3′) were used. The PCR conditions were the same as those used for the amplification of the 8 segments. Amplified products were mixed with the complete genome amplification and subjected to NGS.

Complete genome sequencing was performed on the samples with Ct below 30 via next-generation sequencing. Libraries were prepared with Illumina COVIDSeq Assay (96 Samples) (RUO Version, Document # 1000000126053 v05), using IDT for Illumina-PCR Indexes set 1 or 3. The libraries were pooled and quantified (Qubit DNA HS, Thermo Scientific, Waltham, MA, USA), and their quality was checked (Bio-Fragment Analyzer, Qsep1-Lite, Bi Optic, Santa Clara, CA, USA) before sequencing, which was performed with 10% PhiX control v3, using an iSeq 100 platform and a 300 cycle V2 kit with paired-end sequencing. Viral genome assembly was performed using the INSaFLU bioinformatic tool [12].

### 2.3. Phylogenetic Analysis

Clade determination was performed according to the latest updates of the World Health Organization (WHO), World Animal Health Organization (OIE), and the United Nations for Animal Health, Food and Agriculture Organization (FAO) [13]. An alignment of the 17 HA Venezuelan sequences and 30 reference sequences of previously identified clades obtained from the GISAID and NCBI was performed with MEGA-X [14,15,16]. The phylogenetic tree was created using the best substitution model, following the maximum likelihood method with 1000 Bootstrap replicas, using the sequence A/goose/Guangdong/1/1996 as an outgroup.

For the phylogenetic analysis, sequences from the entire genome of avian influenza H5N1 viruses from northwestern Europe from 2021, and North American sequences from 2022 were obtained from the GISAID and NCBI databases at the beginning of 2023, while the South American sequences were obtained in a period between 2023 and 2024. Sequences were aligned using MEGA-X, and a phylogenetic analysis was performed using IQ-tree with 5000 ultra-fast bootstrap replicates, using the sequence A/barnacle_goose/Netherlands/21025769-002/2021/H5N1 as an outgroup. Visualization and modification of the trees were performed using iTOL [17,18].

### 2.4. Mutation Analysis

The nucleotide sequences of the Venezuelan isolates were aligned and translated into protein in MEGA-X [14]. The presence of mutations was analyzed by comparing the alignments of the Venezuelan samples with the reference sequence A/Vietnam/1203/2004. For segments 6 and 8, the reference sequence A/goose/Guangdong/1/1996 was also included, since the sequence A/Vietnam/1203/2004 exhibits deletions in these fragments. The HA mutations were reported with the numbering described for H5. Mutations that confer adaptations to the H5N1 virus to infect and reproduce in mammals were identified in the literature. The Inf_Pel3 sample was also subjected to analysis on the FluPhenotype server [19] to check for the presence of other mutations. Additionally, protein alignment of the 255 reference HA sequences was performed using MEGA-X. The 325–330 segment of the protein was analyzed, corresponding to the polybasic furin recognition motif, a determining factor in the pathogenicity of avian influenza viruses [20,21,22]. Possible glycosylation sites were analyzed by analyzing the protein sequence of Inf_Pel3 in Expasy-ScanPROSITE [23]. The alignment was built in MultAlin [24].

### 2.5. Docking Analysis

A representative sequence was selected for each South American viral lineage (A/Pelican/Venezuela/Pel3/2022/EPI ISL 16013752, A/chicken/Colombia/Choco/3504/2022, and A/Peru/LAM-002/2022/EPI ISL 16249681), as well as for the Eurasian viral lineage (A/white-tailed eagle/Iceland/2022AI02104/2021/EPI ISL 13245602). Each DNA sequence was translated to protein using MEGA-X [14]. The first 16 amino acids were removed as they were part of the signaling peptide which is cleaved during HA membrane transport. A protein structural model of each of these viral lineages was built using SWISS-MODEL, using as a template the structure of A/duck/Laos/3295/2006(H5N1) (PDB ID 4JUL). Each model was refined and optimized using the YASARA Energy Minimization Server. Model validation was carried out on the SWISS-MODEL Server Tools [25].

For molecular docking between HA and SA receptors, structural analogs of avian and human receptors were downloaded from PubChem. The molecules treated as HA ligands for the analysis were: Alpha-N-acetylneuraminyl-(2,6)-beta-D-galactosyl-(1,4)-N-acetyl-beta-D-glucosamine (PubChem CID 125128) (human receptor analog) and N-acetylalpha-neuraminyl-(2,3)-beta-D-galactosyl-(1,4)-N-acetyl-beta-D-glucosamine (PubChem CID 44611401) (avian receptor analog), as previously reported [26].

Coordinates of the binding site were obtained for each HA protein model using AutoDockTools 1.5.7. Docking was performed for each HA model with the avian and the human receptors respectively, using AutoDock, obtaining 20 docking modes in each run. Up to 5 runs were performed for each docking analysis. Visualization of the docking was performed in PyMOL, where the best interaction model was selected. Binding energy was obtained in each docking run with AutoDock, and assigned after visualization [27].

Additional docking analyses were performed with the HA protein structure and human and avian SA receptor analogues, respectively, using the structures of A/goose/Guangdong/1/1996(H5N1) (PDB ID 4MHI) representative of clade 0, and A/duck/Laos/3295/2006(H5N1) (PDB ID 4JUL) representative of clade 2.3.4. Protein sequence alignment was performed on the MultAlin 5.4.1 webpage (Multiple sequence alignment with hierarchical clustering) [24].

## 3. Results

### 3.1. H5N1 Venezuelan Samples

A total of 67 samples were collected from November 2022 to April 2023 in Venezuela. Two types of samples were analyzed:

(1) Tracheal or cloacal swabs of the dead birds collected along the coast (in a few cases, also organs after autopsy: collection was performed in the best-preserved birds);
(2) Active surveillance in poultry, where no case of influenza H5N1 was detected.

**Figure 1:**
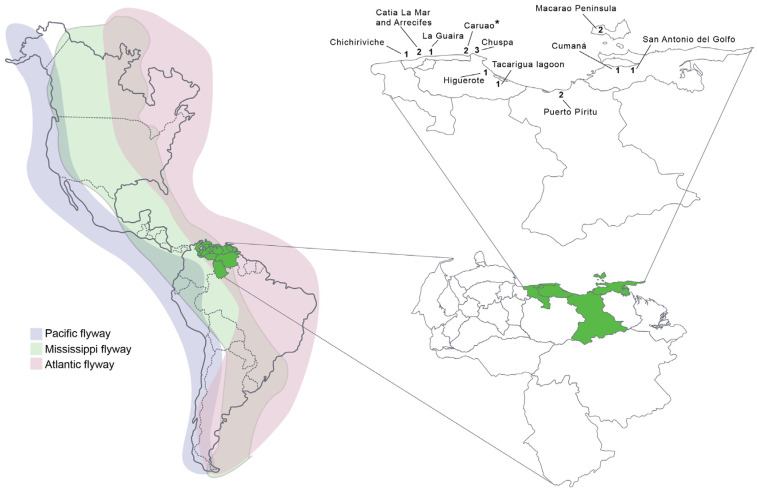
Localities of the H5N1 positive cases in dead birds found along the Venezuelan coast. Numbers indicate the number of infected birds. For many of the other samples tested along the coast, negative results might be due to poor conservation of samples before arriving at the laboratory. * One sample was from a vulture.

A total of 20 samples (19 from Pelicans (*Pelecanus occidentalis*) and one from a vulture (*Coragyps atratus*)) were found to be positive for influenza H5N1, from five states of the country (Figure 1 and Table 1). All of the positive samples were collected along the coast of the country.

From these 20 positive samples, almost complete genome sequences could be obtained for 17 of them: all of the sequences for segments 4 to 8, 12 for segment 1, 13 for segment 2, and 14 for segment 3 (Table 1). The other three sequences exhibited low viral load (Ct over 35 on qRT-PCR), and sequencing was unsuccessful. The very low frequency of sequencing success in samples with Ct over 35 has been described previously [28].

### 3.2. Phylogenetic Analysis

**Figure 2:**
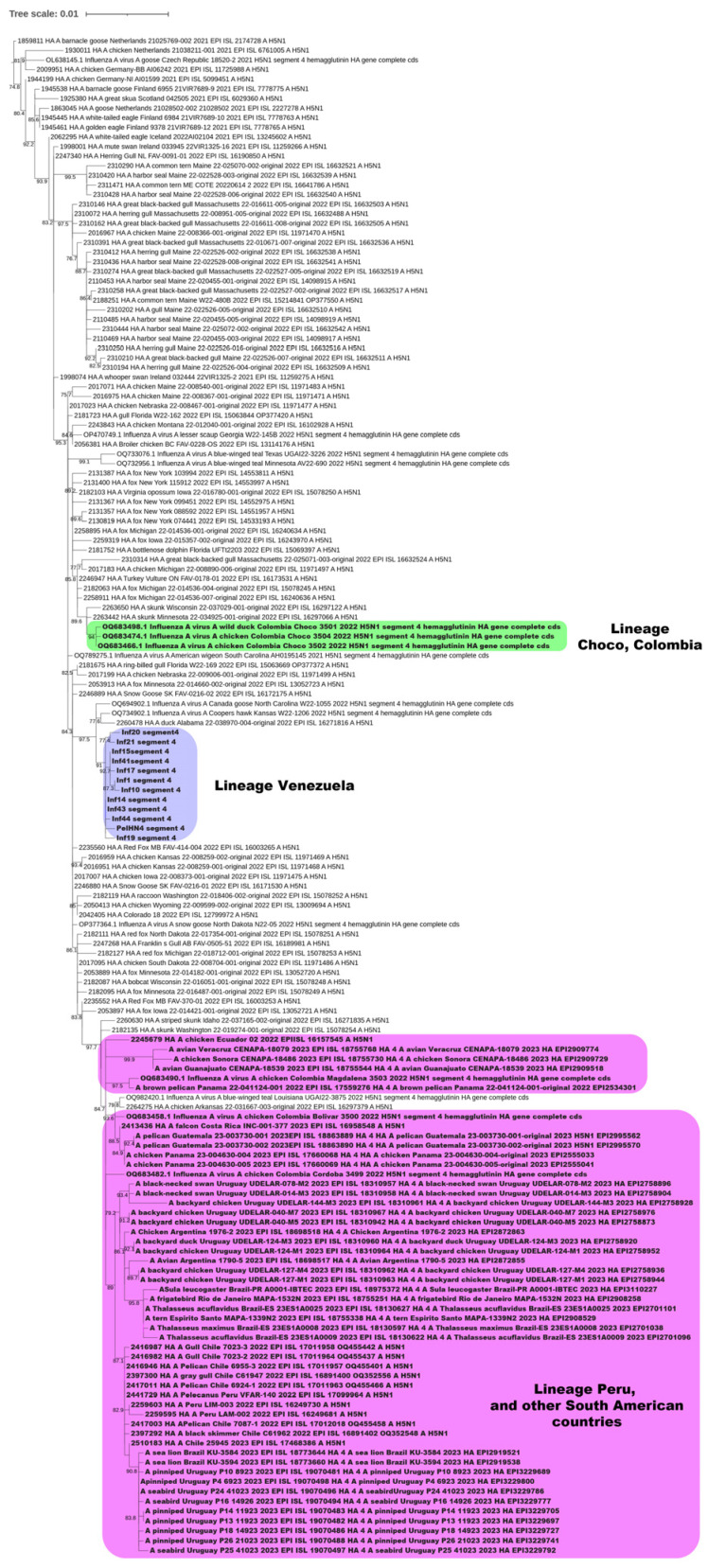
Phylogenetic analysis of the hemagglutinin gene of influenza isolates. ML tree with 5000 replicates using the model GTR + F + G4. All of the sequences are H5N1 clade 2.3.4.4b. Blue sequences are from Venezuela, green ones are from Choco, Colombia, and the pink sequences are from all of the other South American localities. All of the sequences in this phylogenetic tree are of Eurasian origin.

**Figure 3:**
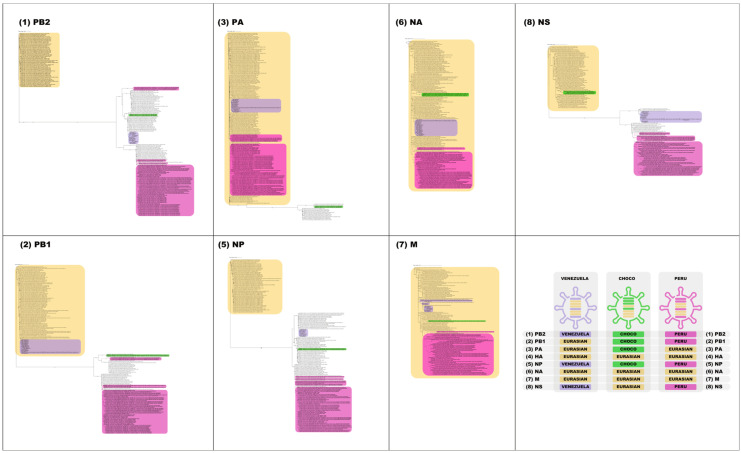
Phylogenetic analysis of the 7 other gene segments of influenza H5N1 (other than H) that circulated in Venezuelan birds in the 2022–2023 outbreak. ML tree with 5000 replicates using the model GTR + F + G4. Blue sequences are from Venezuela, green ones are from Choco, Colombia, and the pink sequences are from all of the other South American localities. Sequences in yellow in the virions and the yellow boxes correspond to a Eurasian origin. (4) HA from Figure 2.

Phylogenetic analysis of the HA gene confirmed the circulation of the H5 2.3.4.4b clade in Venezuela (Figure 2). The analysis of the 8 genomic segments allowed us to confirm that the South American sequences could be divided into three lineages (Figure 2 and Figure 3):

(1) The Chocó-Colombia sequences, in green, share a lineage with North American sequences from Michigan, Minnesota, Massachusetts, Wisconsin, New York (United States), and Ontario (Canada); these sequences correspond to the Atlantic/Mississippi bird flyway genotype B1.2-like [29].
(2) The Venezuelan sequences, in blue, share a lineage with North American sequences from North Carolina, Kansas, and Alabama (United States), derived from the Atlantic flyway. This genotype is composed of PB2, NP, and NS genes from an American lineage and PB1, PA, HA, NA, and MP from the Eurasian one. These isolates were previously described and named Min-Ven by Ospina-Jimenez et al. [29], also associating Venezuelan isolates with viruses from Kansas, New York, Florida, and Canada. This genotype has been reported as a minor group in North America, tracing the American genes to three strains: A/Mallard/Alberta/175/2021(H4N6) with 98.8% identity in PB2, A/Mallard/Alberta/357/2022(H3N8) with 98.9% identity in NP, and A/blue-winged teal/Guatemala/CIP049-H189-19/2019(H14N4) with 99% identity in NS [29].
(3) The rest of the South American sequences include sequences from Ecuador, Peru, Colombia, Brazil, Uruguay, Argentina, and Chile, which share a lineage with the North American sequences from Idaho, Washington, Iowa, Louisiana, Arkansas (United States), and Manitoba (Canada), which correspond to the Pacific flyway, genotype B1.1-like [29].

It is interesting to note that the lineage found in birds from Venezuela was not found in any other country of South America, including Brazil, Argentina, and Uruguay, which share a common flyway (Atlantic). However, the sequences of each genomic segment were closely related to sequences from North American isolates, suggesting that no further reassortment occurred in the country.

In order to analyze the presence of reassortment in the Venezuelan isolates, phylogenetic analysis was performed on the remaining seven genomic segments (Figure 3). For each segment, the Venezuelan isolates grouped together, and three lineages were similarly observed for the Latin American sequences. Then, no further reassortments were observed in the Venezuelan isolates, except those that occurred previously for the Min-Ven genotype.

The 3 lineages circulating in South America contain several segments of Eurasian origin. Segments 4 (Figure 2), 6, and 7 displayed a Eurasian origin for the 3 lineages. In addition, segments 2 and 3 for the Venezuelan lineage, segment 8 for the Choco sequence, and segment 3 for the other lineage were also of Eurasian origin (Figure 3).

### 3.3. Analysis of Mutations Involved in Pathogenesis and Mammal Adaptation

**Figure 4:**
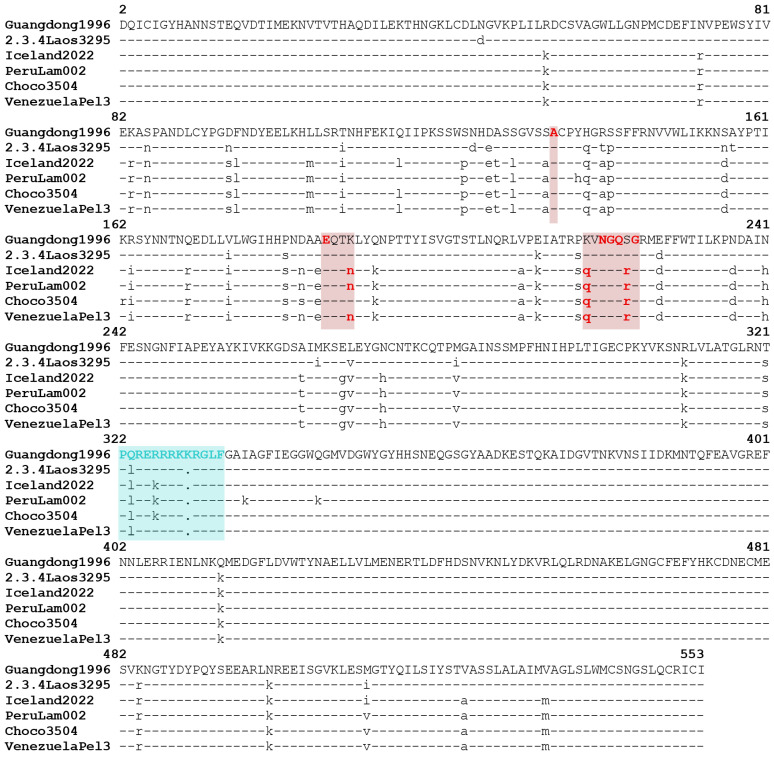
Amino acid alignment of the hemagglutinin protein of the South American lineages. The cyan box highlights the Furin recognition peptide. Red boxes show the amino acids for which some mutations have been shown to increase the affinity to the α2,6-linked sialic acid receptor.

The hemagglutinin of the isolates from the 2021–2023 outbreak of influenza H5N1 clade 2.3.4.4b displayed more than 99% homology between them, 90% homology with the ancestral Guangdong isolate and 93% homology with the 2.3.4 clade. They share the mutation S223R, which has been described as being able to increase the affinity for the human receptor, when present with the other mutations S123P, N193K, and K218Q [30], with it being the last one present in the isolates from the clade 2.3.4.4b (Figure 4). Dadonaite et al. identified seven amino acids for which some substitutions were shown to increase the affinity to the α2,6-linked sialic acid receptor: A137, E190, N193, N224, G225, Q226, and G228 (the numbering of amino acids in the text is according to the cited reference, based on the mature H3 hemagglutinin numbering) [31]. All of these amino acids were conserved among the 3 South American genotypes, except for the N193K found in the Choco isolate (A134, E187, N190, N220, G221, Q222, and G224 in Figure 4, based on the mature H5 numbering). The furin recognition site also displayed some minor polymorphisms among the different isolates, although the differences were mainly conservative (Figure 4).

### 3.4. Docking Analysis

**Figure 5:**
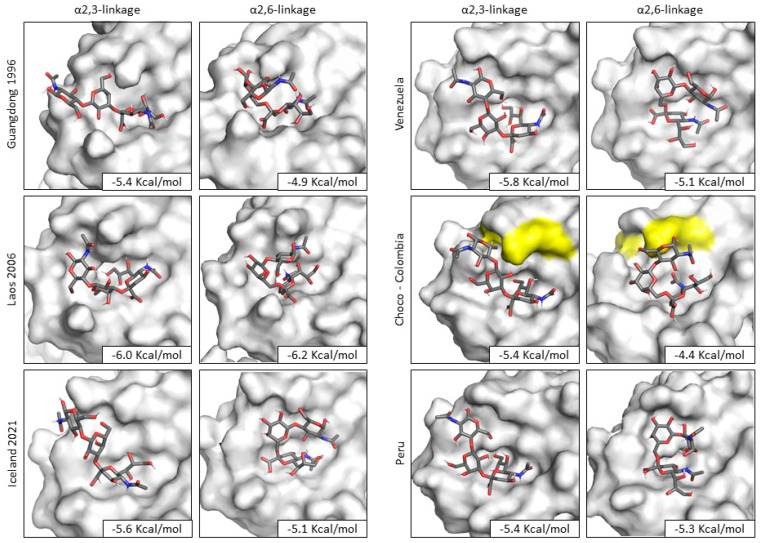
Docking analysis of H5N1 representative South American lineages inside the clade 2.3.4.4b. The amino acid changes in the binding site of the Choco lineage are shown in yellow.

The docking analysis between the hemagglutinin and the sialic acid with avian α-2,3 and human α-2,6 bonds was performed; for this, the hemagglutinin protein of one representative sequence of each South American lineage of clade 2.3.4.4b was modeled three-dimensionally and structurally optimized. For the analysis, two crystallized hemagglutinin structures were retrieved from the PDB and selected as controls, which are clade 0 hemagglutinin from Guangdong in 1996 and the one from Laos 2006 belonging to clade 2.3.4. The results of docking are shown in Figure 5.

Sialic acid binds to the hemagglutinin between loops 130 and 220 and helix 190. The conformation of the avian sialic acid receptor assumes an extended conformation, while human sialic acid assumes a bent conformation. The hemagglutinin of the viruses isolated in Chocó exhibits a change of amino acid N190K, which makes the binding site narrower and makes binding of the human sialic acid receptor even more difficult, which is represented by the lower binding energy.

The interaction between the hemagglutinin of Venezuelan isolates and sialic acid presented a greater number of hydrogen bonds in the case of the binding with the avian receptor; it was also observed that the main interaction occurs in loop 220, which presents amino acids associated with the preference of the virus to interact with avian receptors.

## 4. Discussion

The avian influenza epidemic currently occurring worldwide is unprecedented in its scale. It has led to massive mortality events that have affected more than 320 species at the population level, the vast majority of which are aquatic birds, generating serious ecological and conservation problems [32]. On the Peruvian coast alone, the virus killed more than 22,000 wild birds in four weeks, including threatened species such as the Peruvian pelican (*Pelecanus thagus*) and the Peruvian booby (*Sula variegata*) [33]. Even more worrying is the transmission between mammal species already reported in South America. In Chile, there have been more than 4700 cases of infection of animals belonging to 33 different species, including cases of birds and mammals such as sea lions (*Otaria flavescens*) and otters (*Lontra feline*) [34]. Likewise, the first human case of avian influenza in South America was reported in Ecuador, in a nine-year-old girl who was in contact with infected poultry [35]. Subsequently, another human infection occurred in Chile, where the patient presented with severe symptoms of the disease [36]. Although these outbreaks began in 2021, in recent decades, a series of key events have occurred that have led to the emergence and maintenance of this viral clade, such as its rapid evolution, the possibility of generating reassortments, and its ability to infect multiple species [32].

Phylogenetic analysis of the HA gene showed that all of the sequences from the clade 2.3.4.4b circulating in South America share a common ancestor in North America and that these in turn share a monophyletic group with the sequences from northern Europe, as reported previously by several authors [37,38]. As of 19 October 2022, H5N1 outbreaks began to be detected in South America [39]. Phylogenetic studies determined the separation of the South American sequences into three different lineages. This may give indications of different introductions of the virus from the United States and Canada, associated with the migration of birds infected by influenza viruses of different genotypes along the various migratory routes [40,41]. In fact, the sequences from the department of Chocó in Colombia and the Venezuelan sequences are related to sequences from the Central–Eastern states of the USA and Canada, while the sequences from Brazil, Ecuador, Peru, Chile, and other localities in Colombia seem to be more related to sequences from the Central and Western USA and Canada. This coincides with the migratory routes of the Atlantic and the Pacific, respectively, in agreement with what has been previously reported [41]. The Venezuelan sequences, although related to the other South American ones, formed a distinct lineage without any other South American sequence. A total of 25 entry sites for migratory birds have been described in Venezuela [42]. Fortunately, no endemic transmission to local birds has occurred inside the country.

In addition to geographical barriers, the other variable to consider is the different species of infected birds that carry the virus. In the case of the outbreak that occurred in Venezuela, the largest number of affected animals corresponds to pelicans. King et al. 2013, reported that the maximum distance traveled by a pelican is 900 km [43]; thus, it is unlikely that the pelicans that died in Venezuela were infected outside the Venezuelan coast. The blue-winged duck (*Spatula discors*) may be one of the species that can migrate long distances between North and South America along the Atlantic migratory route; thus, it could become infected during the mating and breeding period throughout the northern United States and spread the virus to Central America, the Caribbean, and Northern South America during the wintering period [44,45].

The great variability of influenza viruses found in the Americas is largely due to the reassortment of the viral genome. However, certain genotypes may not form efficiently due to incompatibilities between vRNA packaging signals. Furthermore, most reassorted viruses formed from divergent parental viruses are usually outcompeted by one or both viral parents due to incompatibilities between the reassorted proteins [46], as well as bottlenecks determined when leaving the cell, due to the mucociliary elimination system and selection by antibodies of the host immune system. This immune barrier is likely to substantially reduce the population of newly generated variants, and only a minority of the virus progeny can successfully infect other cells [47].

Following the first outbreaks in Newfoundland and the South Atlantic states of the United States in December 2021, Kandeil et al. described a rapid intercontinental expansion of clade 2.3.4.4b towards western North America [48]. During this process, HPAI viruses that originated in Europe recombined with LPAI viruses from North American wild birds, producing reassortments with various gene mixtures and generating at least four genotypes of H5N1 circulating in the country (i.e., the genotype with all segments of Eurasian origin and three genotypes with reassortment). In the beginning, all genotypes generated from reassortments maintained the parental Eurasian origin in their segments 4, 6, 7, and 8, but they had different combinations of Eurasian or North American origin in the polymerase complex (PB2, PB1, and PA genes) and NP gene segments. Then, some viruses also incorporated segment 8 from LPAI North American viruses. In fact, Youk et al. reported six main genotypes in the United States, the ancestral genotype of Eurasian origin, and five additional genotypes originating from genomic reassortments between ancestral viruses and different subtypes of LPAI viruses; these new viruses in turn also generated reassortments, further varying the constellations of circulating influenza genes [49]. Proteins from the North American lineage of viruses had no markers associated with increased virulence in mammalian hosts, retained specificity to the avian receptor, and all viruses remained antigenically homogeneous. However, the manifestations of the disease varied in the animals studied, with viruses with genotypes containing a greater number of segments of North American origin exhibiting greater virulence with neurological implications in mammalian models [48].

The viral sequences from the outbreak observed in Venezuela were homogeneous in terms of not displaying further reassortment in any of their segments. However, the three Latin American clades present different genotypes compared to one another, possibly generated by reassortment events that occurred in North America between different viruses, as previously reported for the viruses of the 2022–2023 outbreak present in different South American countries [41,50].

The phylogenetic analysis of all of the segments showed that in the three South American lineages, segments 4, 6, and 7, which code for hemagglutinin, neuraminidase, and matrix proteins, respectively, have a Eurasian origin; they share similarities with North American sequences. Additionally, segments of Eurasian origin were present in segments 2 and 3 for the Venezuelan lineage, while segments 1, 5, and 8 exhibited an American origin, as previously reported [41]. The Chocó-Colombia sequences exhibited segment 8 of Eurasian origin and segments 1, 2, 3, and 5 of North American origin, as previously reported [49]. Finally, the sequences of segment 3 for the Chile and Peru lineage had a Eurasian origin, and segments 1, 2, 5, and 8 had a North American one, in agreement with previous reports [41,50]. That is, the Venezuelan sequences harbor a greater number of segments of Eurasian origin, while those from the rest of South America present an equal number of segments of Eurasian and North American origin, the latter being associated with greater pathogenicity [48].

However, due to the large number of reassortment events reported in North America, it is difficult to ascertain the origin of each genomic constellation of the viruses found in South America. After the phylodynamic reconstruction carried out by Youk et al., 2023, from the data obtained by Leguia et al., 2023 and Ruiz-Sáenz et al., 2023 and what was analyzed in this project, we can infer that the South American viruses originated from viruses with a genotype of Eurasian origin that spread along the migratory route of the Atlantic to North Carolina and South Carolina and then south to Florida along the Atlantic coast and to the central United States via the Mississippi and Central Flyways [40,41,49].

Most of the HA sequences from the clade 2.3.4.4b exhibited a polybasic site consisting of seven amino acids with the sequence PLREKRRKR/G in the protease cleavage site, one residue less and a different amino acid composition than the sequence of the Guangdong virus lineage. Analysis of HA polybasic motifs revealed a predilection of H5 HPAI viruses to evolve and harbor seven basic residues in the polybasic motif. Segments of intermediate length (3 to 4 residues) are rapidly replaced by extended forms. In this way, viruses with polybasic sites with a medium length are found at a lower proportion in nature [51].

Residues at positions P1, P6, and P4 are, in this order, the most important amino acids of the hemagglutinin polybasic segment for interaction within the furin pocket, with arginine being the preferable amino acid in all three positions, with residues P1 and P4 also being the most conserved and presumably the most functionally important [51,52]. All three South American lineages have arginine at positions P1 and P4. But Venezuelan samples also presented an arginine (R) instead of a lysine (K) in the amino acid sequence of the polybasic segment at the P5 position. Lee et al., 2006 did not find differences in the virulence of another virus subtype (H7N2) mutated at the P5 position [53]. Guo et al., [52] reported that the loss of the lysine at the P3 position of the polybasic segment of the Guangdong virus, which perfectly coincides with the amino acid sequence of the polybasic segment of the Venezuelan viruses, determines a slightly weakened binding energy and a different substrate conformation within the furin binding pocket. This fact, the substrate not fitting well, could determine a decrease in the pathogenicity of these viruses.

In all of the isolates analyzed from clade 2.3.4.4b, a glutamic acid (E), which is an acidic residue, was present at position P6. An acidic residue on P6 seems counterintuitive since the residue would impart repulsive forces when binding to furin. On the other hand, this motif at the cleavage site may be indicative of isolates that are about to complete the extension of the polybasic motif; the incorporation of another basic residue would “push” the suboptimal acidic residue from the P6 to P7 position, thus increasing interactions between HA and protease [54]. This phenomenon was observed only in one sequence A/fox/NewYork/107242/2022/(H5N1), where an arginine (R) was inserted at the P5 position, displacing the lysine (K) to P6 and the glutamic acid (E) to P7.

**Figure 6:**
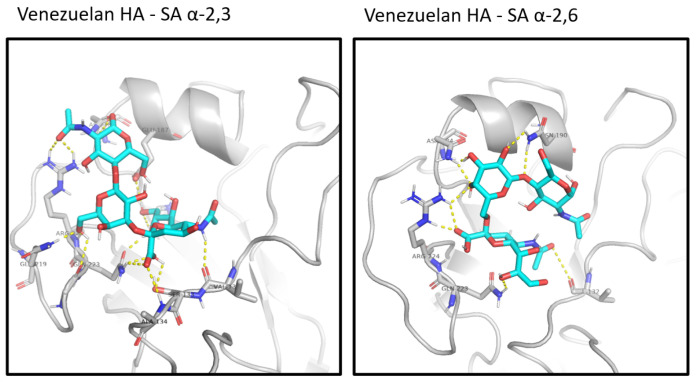
Venezuelan virus hemagglutinin binding site showing hydrogen bonding with the avian and human forms of sialic acid, respectively. A total of 14 H-bonds are formed between avian sialic acid and the following amino acids of HA: V132, S133, A134, H180, N184, E187, Q219, G222, R224. A total of 8 H-bonds are formed between human sialic acid and the following amino acids of HA: V132, N184, N190, Q223, and R224.

The analysis of other mutations associated with pathogenicity or mammalian adaptation showed the presence of several ones when compared to the ancestral Guangdong sequence. However, Venezuelan isolates maintained the QRG motif at position 222–224 of the hemagglutinin binding site, which determines its preference for interaction with the avian receptor [20,21,55]. Additionally, it presents the amino acid combination K218Q/S223R, which increases its binding capacity to specific fucosylated sialoside receptors in the intestinal epithelium of birds [30]. The docking analysis showed that the interaction of hemagglutinin with the avian SA α-2,3 receptor presented 14 hydrogen bonds compared to the human SA α-2,6 receptor which only presented 8 hydrogen bonds. This corroborates an increased affinity towards the avian receptor of the Venezuelan isolates (Figure 6).

The docking analysis also showed that the three lineages circulating in South America exhibited similar affinity values to the host receptors and maintained a higher affinity for the avian one. In fact, it can be observed that the receptor binding site of the Chocò isolates also exhibits similarities with the receptor binding site of the Guangdong isolate since they share a lysine (K) at position 190, which makes the cavity of the binding site narrower and therefore the binding of the human receptor in the folded conformation is disadvantaged [56].

On the other hand, the receptor binding site of the Pacific isolates has a structure similar to that of the Venezuelan isolates, in terms of amino acid composition and therefore structurally. However, it has been observed that these isolates have been much more pathogenic, affecting a large number of mammals, possibly due to the presence of mutations in PB2 such as D701N and Q591K that were found in sea lions in Peru and Chile; these mutations are involved in increased viral transcription and replication [35,41].

The similarities between affinity values do not imply that avian influenza viruses will infect humans, although hemagglutinin could interact with the α-2,6 receptor; in the folded conformation, saccharides positioned after N-acetylglucosamine can generate steric clashes with the HA and prevent interaction [57].

## 5. Conclusions

The unprecedented pathogenic outbreak of avian influenza in South America was associated with the circulation of three different lineages, which maintain a lower affinity for the mammalian receptor.

## Figures and Tables

**Table 1:** Sequenced samples of influenza H5N1 viruses from Venezuela.

ID	State	Locality	Date	Animal	Segments Sequenced	GISAID
Pel3	Anzoátegui	Puerto Píritu	25/11/2022	Pelican	1–8	EPI_ISL_16013752
PelHN4	Anzoátegui	Puerto Píritu	25/11/2022	Pelican	1–8	EPI_ISL_16013753
Inf1	Miranda	Tacarigua	7/12/2022	Pelican	1–8	EPI_ISL_18909019
Inf3	Miranda	Higuerote	7/12/2022	Pelican	4–8	EPI_ISL_16631939
Inf9	La Guaira	Chuspa	12/12/2022	Pelican	1–8	EPI_ISL_16631973
Inf10	La Guaira	Río Chuspa	12/12/2022	Pelican	1,3–8	EPI_ISL_18931240
Inf11	La Guaira	Río Chuspa	12/12/2022	Pelican	2,4–8	EPI_ISL_18931241
Inf12	La Guaira	Río Chuspa	12/12/2022	Pelican	-	NA
Inf13	Nueva Esparta	Macanao	16/12/2022	Pelican	1–8	EPI_ISL_16701840
Inf14	Nueva Esparta	Macanao	16/12/2022	Pelican	1–8	EPI_ISL_18931268
Inf15	La Guaira	Guaira beach	20/12/2022	Pelican	3–8	EPI_ISL_18931269
Inf16	La Guaira	Guaira beach	20/12/2022	Pelican	-	NA
Inf17	La Guaira	Catia La Mar	20/12/2022	Pelican	2,4–8	EPI_ISL_18931270
Inf 18	La Guaira	Naiguata	20/12/2022	Pelican	-	NA
Inf19	Sucre	Cumaná	20/12/2022	Pelican	1–8	EPI_ISL_18931271
Inf20	La Guaira	Caruao	23/12/2022	Pelican	1–8	EPI_ISL_18931272
Inf21	La Guaira	Caruao	23/12/2022	Vulture	1,3–8	EPI_ISL_18931273
Inf41	La Guaira	Chichiriviche	11/1/2023	Pelican	1–8	EPI_ISL_18931274
Inf43	La Guaira	Arrecife	11/1/2023	Pelican	2–8	EPI_ISL_18931275
Inf44	Sucre	Golfo	27/1/2023	Pelican	1–8	EPI_ISL_18931276

NA: sequence not available: unsuccessful sequencing attempt, probably due to a high Ct value (low viral load hampering sequencing) on qRT-PCR.

## Data Availability

The complete genome sequences have been deposited in the GISAID database, with the accession numbers listed in Table 1.
